# An Infection-Based Murine Model for Papillomavirus-Associated Head and Neck Cancer

**DOI:** 10.1128/mBio.00908-20

**Published:** 2020-05-12

**Authors:** Tao Wei, Darya Buehler, Ella Ward-Shaw, Paul F. Lambert

**Affiliations:** a McArdle Laboratory for Cancer Research, University of Wisconsin School of Medicine and Public Health, Madison, Wisconsin, USA; b Department of Pathology and Laboratory Medicine, University of Wisconsin School of Medicine and Public Health, Madison, Wisconsin, USA; Princeton University

**Keywords:** MmuPV1, head and neck cancer, papillomavirus

## Abstract

Human papillomavirus (HPV) is the most common sexually transmitted pathogen, and high-risk HPVs contribute to 5% of human cancers, including 25% of head and neck squamous cell carcinomas (HNSCCs). Despite the significant role played by HPVs in HNSCC, there is currently no available *in vivo* system to model the process from papillomavirus infection to virus-induced HNSCC. In this paper, we describe an infection-based HNSCC model, utilizing a mouse papillomavirus (MmuPV1), which naturally infects laboratory mice. Infections of the tongue epithelium of two immunodeficient strains with MmuPV1 caused high-grade squamous dysplasia with early signs of invasive carcinoma over the course of 4 months. When combined with the oral carcinogen 4-nitroquinoline-1-oxide (4NQO), MmuPV1 caused invasive squamous cell carcinoma (SCC) on the tongue of both immunodeficient and immunocompetent mice. These tumors expressed markers of papillomavirus infection and HPV-associated carcinogenesis. This novel preclinical model provides a valuable new means to study how natural papillomavirus infections contribute to HNSCC.

## Abstract (precis)

The species specificity of papillomavirus has limited the development of an infection-based animal model to study HPV-associated head and neck carcinogenesis. Our study presents a novel *in vivo* model using the mouse papillomavirus MmuPV1 to study papillomavirus-associated head and neck cancer. In our model, MmuPV1 infects and causes lesions in both immunodeficient and genetically immunocompetent strains of mice. These virally induced lesions carry features associated with both HPV infections and HPV-associated carcinogenesis. Combined with previously identified cancer cofactors, MmuPV1 causes invasive squamous cell carcinomas in mice. This model provides opportunities for basic and translational studies of papillomavirus infection-based head and neck disease.

## INTRODUCTION

Papillomaviruses are species-specific, nonenveloped, double-stranded small (∼8-kbp) DNA viruses. Human papillomavirus (HPV) is the most common sexually transmitted pathogen in the United States (1, 2), and high-risk HPVs such as HPV16, -18, and -33 cause 5% of human cancers (3, 4). Approximately 25% of head and neck squamous cell carcinomas (HNSCCs) are associated with HPV infections, and the percentage of HNSCCs associated with HPVs has increased over the past several decades (5–7). Despite the significant influence of HPVs on head and neck carcinogenesis, a tractable, papillomaviral infection-based model for HNSCC is currently unavailable.

The species specificity of papillomavirus has limited the development of an infection-based animal model to study how viral infection contributes to cancer development (8, 9). In the absence of such a model, transgenic mouse models have been developed. Among these is the keratin 14 promoter (K14)-HPV16E6E7 transgenic mouse, in which the expression of the HPV16 viral oncogenes E6 and E7 is targeted to the basal layer of the stratified epithelium. Such models have been extremely valuable for defining the contributions of viral oncoproteins and host factors to HPV-associated tumorigenesis, including HNSCC (10–14). Tumor graft models using mouse tonsil epithelial cells transformed by HPV16 oncogenes and activated Ras (15) have provided additional insight into the role of host immunity in the response to radio- or chemotherapy (16) and the response of recurrent/metastatic disease to drug therapies (17). However, none of these models recapitulate processes that arise from natural HPV infections of normal human tissues. Other animal models of papillomavirus infection use rabbits or dogs infected with cottontail rabbit papillomavirus (CRPV) or canine oral papillomavirus (COPV), respectively (9, 18). These animal species are not easily manipulated genetically, making it difficult to study the role of host factors, including the host immune system. However, the recent discovery of a mouse papillomavirus, MmuPV1, that infects laboratory mice (19–21) has opened the door to generating infection-based models for papillomavirus-associated pathogenesis in an animal that is highly tractable and can be genetically manipulated. While initially found to cause cutaneous warts in immunodeficient mice, MmuPV1 also causes cutaneous warts in immunocompetent strains with or without alterations to their immune system (19, 22–27). Importantly, MmuPV1 has a wide tissue tropism: it infects not only cutaneous epithelia but also sites typically implicated in the sexual transmission of HPV, including the mucosa of the female and male genitalia, the anus, and oropharyngeal sites (28–34). We have previously shown that immunocompetent mice experimentally infected with MmuPV1 in their female reproductive tract developed cervical and vaginal cancers (28), much like high-risk HPVs in humans. This observation led us to investigate whether MmuPV1 infections of mucosal epithelia in the head and neck region can also progress to carcinoma.

In this study, we show in two immunodeficient strains of mice, *Nod SCID Gamma* (NSG) and *Fox^nu/nu^* (nude), that MmuPV1 can infect the squamous epithelium of the dorsal surface of the tongue. The virus causes visible papilloma-like exophytic lesions that spontaneously progress to high-grade dysplasia. We also detected secondary infections arising at the base of the tongue, a site where HPV-associated HNSCCs occur in humans, although mice lack the equivalent to the tonsillar crypt epithelium. Upon exposure to an oral carcinogen, 4-nitroquinoline-1-oxide (4NQO) (14, 35, 36), MmuPV1-infected nude mice developed invasive SCCs. These carcinomas express markers of MmuPV1 infections and biomarkers commonly found in HPV-associated cancers. We also observed upregulated levels of cytokeratin 17 in MmuPV1-induced oral tumors, a host gene recently identified to mediate papillomavirus-induced immunosuppression (26). We extended our studies to genetically immunocompetent *FVB* mice and found that MmuPV1 also infects the tongue. When mice were treated with 4NQO, we observed that an increased incidence and severity of neoplastic progression in these infected mice were evident compared to mock-infected mice, including the development of invasive SCCs. These studies are the first to describe a tractable, infection-based model for papillomavirus-induced HNSCC in laboratory mice. This new model will open the door to gaining new insights into papillomavirus infection-mediated development of HNSCC.

## RESULTS

### Development of an MmuPV1 oral infection model in immunodeficient mice.

**FIG 1:**
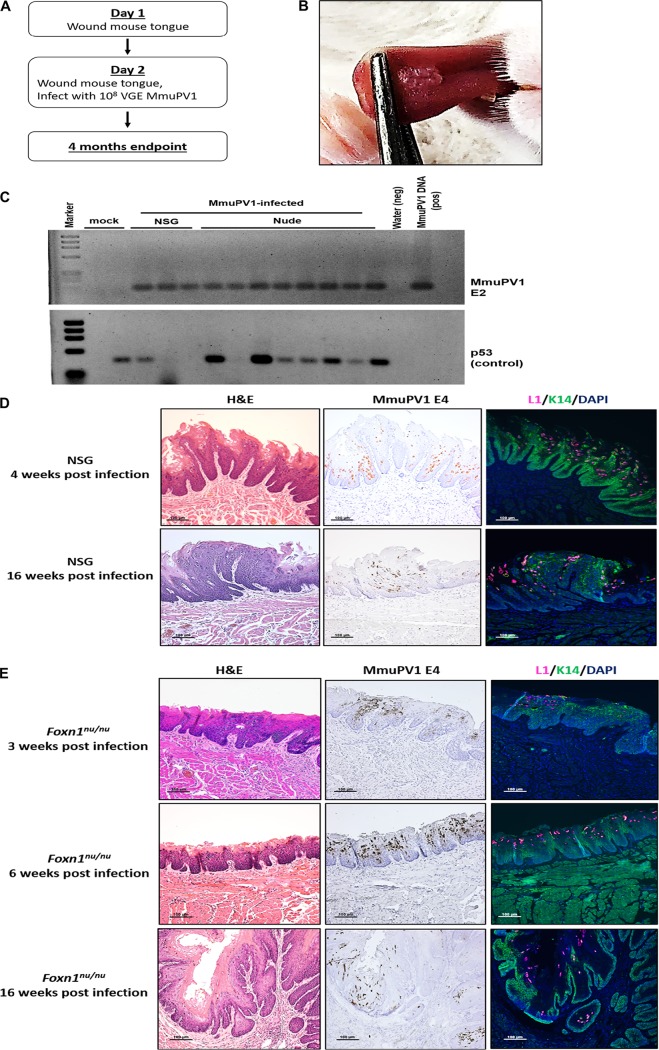
MmuPV1 infects and causes high-grade disease on the tongue of immunodeficient mice. (A) Method for tongue infections in immunodeficient mice. (B) Overt exophytic papilloma-like lesion observed on the tongue of NSG mice at the end of 4 weeks postinfection. (C) Presence of virus detected by performing PCR on the MmuPV1 E2 gene in oral swab samples collected from infected NSG mice at 4 weeks postinfection (mock, *n* = 2; MmuPV1 infected, *n* = 3) or from infected *Foxn1^nu/nu^* mice at 3 weeks postinfection (*n* = 8). The presence and quality of the extracted DNA were confirmed by performing PCR on the cellular p53 gene. (D) Representative images showing that in NSG mice, MmuPV1 can cause papilloma formation at 4 weeks postinfection that progresses into high-grade dysplasia by the end of 16 weeks. (E) Representative images of disease progression in MmuPV1-infected *Foxn1^nu/nu^* mice. The virus infected mice and caused low-grade dysplasia at 3 weeks postinfection. The disease region spread by the end of 6 weeks. At 16 weeks postinfection, the virus alone caused high-grade disease and showed early signs of carcinoma.

To develop an infection model in the murine oral tract using MmuPV1, we first infected the tongues of immunodeficient strains of mice. We adapted an oral infection method that was previously used by Cladel and colleagues (31, 32) (Fig. 1A). Mice were wounded and infected as described previously and monitored over 16 weeks for papilloma development.

Overt exophytic papilloma-like lesions appeared at the sites of infection by 4 weeks postinfection in NSG mice (Fig. 1B), an immunodeficient strain that lacks responses of T cells, B cells, and natural killer cells (37). We tracked the presence of virus on the tongue by collecting oral swab samples and performing PCR to detect MmuPV1 DNA (E2 gene). Another PCR was performed on the cellular p53 gene to confirm the presence and quality of DNA extracted from the swab samples. All MmuPV1-infected NSG mice were positive for MmuPV1 DNA, whereas no viral DNA was detected in the mock-infected mice (Fig. 1C).

Tissues from two MmuPV1-infected NSG mice were harvested at either 4 or 16 weeks postinfection and analyzed histopathologically (Fig. 1D). A virus-induced papilloma-like exophytic lesion was observed in the infected tongue at 4 weeks postinfection, with the presence of koilocytes, a hallmark of papillomavirus infection (28, 38), and low-grade dysplasia. By 16 weeks postinfection, high-grade dysplasia had developed at the infection site. *In situ* hybridization confirmed the presence of viral E4 transcripts in both lesions from both 4 and 16 weeks postinfection. Both lesions also tested positive for the viral capsid protein L1, indicating the establishment of the productive phase of the MmuPV1 life cycle.

We also infected nude mice, *Foxn1^nu/nu^*, another immunodeficient strain that lacks functional T cells (39), using the same method (Fig. 1A). All infected mice tested positive for MmuPV1 at 3 weeks postinfection by PCR analysis of oral swab samples (Fig. 1C). Tissues from infected mice were harvested at 3, 6, and 16 weeks postinfection and analyzed histopathologically (Fig. 1E). At 3 weeks postinfection, MmuPV1 induced low-grade squamous dysplasia at the site of infection. The size of the infected area expanded by week 6. By 16 weeks postinfection, most of the tongue epithelium was infected with MmuPV1. The expansion of MmuPV1 infection over time is particularly evident in low-magnification images (see Fig. S1A and B in the supplemental material). We also observed high-grade dysplasia/carcinoma *in situ* (CIS) with early signs of invasive carcinoma at 16 weeks postinfection. All lesions tested positive for the MmuPV1 E4 transcript and L1 protein, confirming the establishment of the productive phase of the viral life cycle. Together, these findings demonstrate that MmuPV1 infects the surface epithelium of the tongue in immunodeficient strains of mice and causes high-grade dysplasia.

10.1128/mBio.00908-20.1 FIG S1 Disease progression images of MmuPV1-infected nude mice at low magnification. (A) Serial sections from tongues of mice harvested at 3 weeks (top row), 6 weeks (middle row), or 16 weeks (bottom row) stained with H&E (left column) or subjected to an MmuPV1 E4-specific RNAscope assay (middle column) or immunofluorescence staining (right column) for L1 (red) and keratin 14 promoter (K14) (green) and counterstained with DAPI (blue). At 3 weeks postinfection, the MmuPV1-induced lesion was mostly restrained at the site of infection. By the end of 6 weeks postinfection, the disease region had extended from both ends of the infection site. At 4 months, a large proportion of the infected tongue displayed a high level of abnormality, with high-grade dysplastic features, including some early signs of invasive squamous cell carcinoma. (B) Quantification of the sizes of infected areas. The measurement was performed by using ImageJ (version 1.5k), based on L1 signals as an indicator of the infected area. Each dot represents the size of the infection area from one sample. Download FIG S1, PDF file, 0.4 MB. Copyright © 2020 Wei et al. 2020 Wei et al. This content is distributed under the terms of the Creative Commons Attribution 4.0 International license.

### MmuPV1 cooperates with 4NQO to cause SCC in the oral cavity of immunodeficient mice.

**FIG 2:**
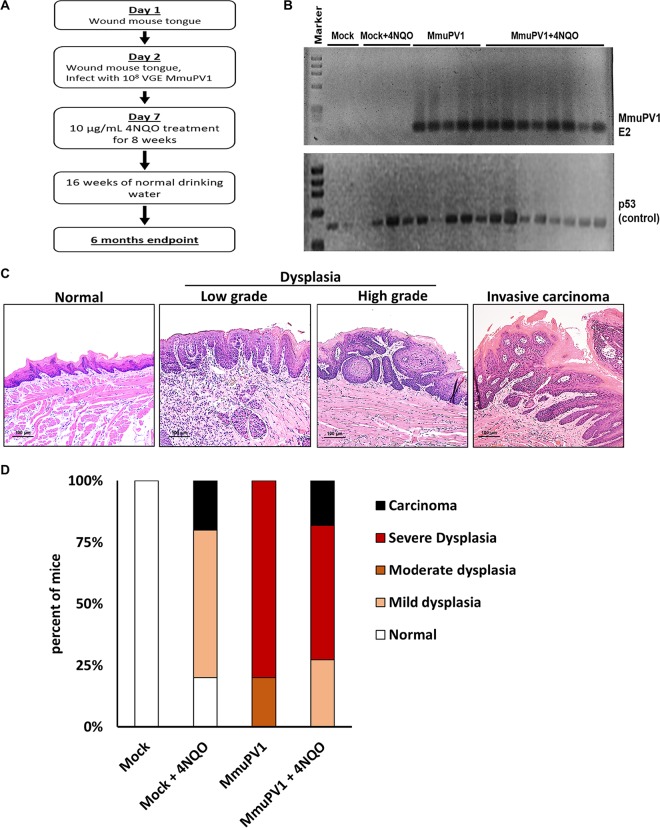
MmuPV1, along with 4NQO treatment, is able to cause invasive carcinoma on the tongue of immunodeficient mice. (A) Method for tongue infections in immunodeficient mice with treatment with the oral carcinogen 4NQO. (B) Presence of virus detected by performing PCR on the MmuPV1 E2 gene in oral swab samples collected from infected NSG mice at 4 weeks postinfection. p53 PCR was performed to confirm the presence of extracted DNA. (C) Representative H&E-stained images of MmuPV1-induced oral disease in different histopathological stages. (D) Disease severity in each experimental group. MmuPV1 infection largely increased the severity of disease in mice. We also observed invasive carcinoma with treatment with 4NQO (*P* = 0.01 for mock versus MmuPV1; *P* = 0.009 for mock versus MmuPV1 and 4NQO [by a two-sided Wilcoxon rank sum test]). Detailed information on each group is shown in Table 1.

Knowing that the virus can establish persistent infections in the tongue that progress to high-grade dysplasia with early signs of invasion, we assessed whether this virus could cooperate with an oral carcinogen, 4NQO, to efficiently cause invasive SCCs. We have previously shown that 4NQO synergizes with HPV16 oncogenes to induce HNSCC (14, 40–44). 4NQO specifically causes cancers in the oral cavity and esophagus when delivered in drinking water (35, 36). The experimental design for this carcinogenesis study is outlined in Fig. 2A and included 4 groups of nude mice, (i) mock infected, (ii) mock infected with 4NQO treatment, (iii) MmuPV1 infected, and (iv) MmuPV1 infected with 4NQO treatment, with 4NQO treatment being initiated at 7 days postinfection (Fig. 2A). The carcinogen was delivered in the drinking water at 10 μg/ml for a period of 8 weeks. The mice were then put back on drinking water without the carcinogen for another 16 weeks to allow cancers to develop. All infected mice tested positive for MmuPV1 DNA at 4 weeks postinfection (Fig. 2B). p53 PCR was performed again as a control for the DNA input in the oral swab samples.

At week 25, the tongues of the mice were harvested, formalin fixed, paraffin embedded, stained with hematoxylin and eosin (H&E), and examined for the presence and degree of dysplasia and invasive carcinoma by a pathologist (D. Buehler) in a blind fashion. The lesions that developed closely recapitulated the appearance of HPV-driven squamous dysplasia and SCCs of the cervicovaginal and anogenital regions in humans (Fig. 2C). The dysplastic lesions are exophytic or flat, with various degrees of koilocytosis and nuclear atypia ranging from mild (low-grade dysplasia) to severe (high-grade dysplasia/CIS). The latter lesions are characterized by a typically acanthotic squamous mucosa with a crowded proliferation of atypical basaloid or dyskeratotic cells with hyperchromatic, overlapping nuclei; irregular nuclear contours; and mitotic activity above the basal one-third of the epithelium. The invasive carcinomas also resemble cervicovaginal/anogenital SCCs, showing stromal invasion with a reactive response, paradoxical maturation in the form of an abundant dense eosinophilic cytoplasm at the invasive front, or single dyskeratotic cells in the stroma.

Detailed scoring results for this study are summarized in Table 1. Infection by MmuPV1 alone significantly increased disease severity (Fig. 2D) (mean score for MmuPV1 versus mock, 2.8 versus 0 [*P* {two sided} = 0.01]), indicating that the virus alone is sufficient to induce dysplastic changes in the oral cavity. However, convincing invasive carcinoma was observed only upon treatment with 4NQO, suggesting that other genetic mutations induced by this carcinogen are important in promoting MmuPV1-driven carcinoma development (Fig. 2D) (mean score for mock plus 4NQO versus mock of 1.4 versus 0 [*P* {two sided} = 0.07]; mean score for MmuPV1 plus 4NQO versus mock, 2.5 versus 0 [*P* {two sided} = 0.009]). Our findings demonstrate that, in combination with a chemical carcinogen, MmuPV1 can cause invasive SCC in the oral cavity.

**TABLE 1:** Disease severity in MmuPV1-infected *Foxn1^nu/nu^* mice^*a*^ with or without treatment with 10 μg/ml 4NQO

Group	Total no. of mice	No. of mice with disease severity				
		Normal	Dysplasia			Invasive carcinoma
			Mild	Moderate	Severe	
Mock	3	3	0	0	0	0
Mock + 4NQO	5	1	3	0	0	1
MmuPV1	5	0	0	1	4	0
MmuPV1 + 4NQO	8	0	3	0	3	2

a All *Foxn1^nu/nu^* mice in this study were females.

### Evidence of virus in MmuPV1-induced oral disease in *Foxn1^nu/nu^* mice.

**FIG 3:**
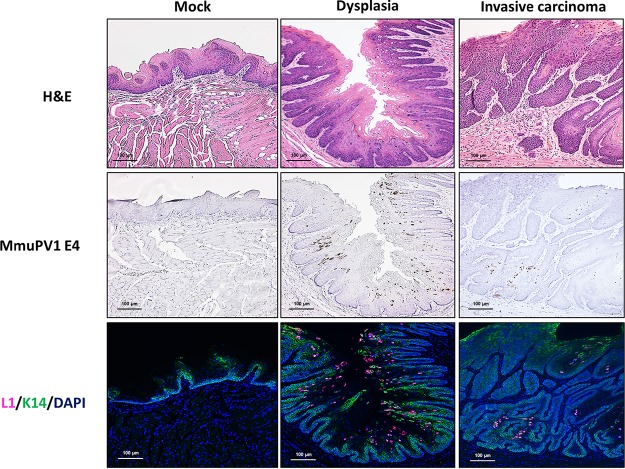
Evidence of virus in MmuPV1-induced oral disease in *Foxn1^nu/nu^* mice. Representative images demonstrate the presence of the MmuPV1 E4 transcript and the capsid protein L1 in sites of dysplasia and invasive carcinoma. Note the reduced level of L1 protein at the carcinoma site.

We examined tongue lesions for the presence of MmuPV1 (Fig. 3). By immunofluorescence, we were able to detect viral L1 in sites of both dysplasia and invasive carcinoma in MmuPV1-infected tongue tissue but not in the mock-infected tongues. MmuPV1 E4 transcripts were also detected in lesions by *in situ* hybridization, in the same L1-positive area. The expression of MmuPV1 L1 is not restricted to the most differentiated layer of cells in the stratified epithelium, which is consistent with previous reports from our laboratory and others (28, 34, 45). Interestingly, we observed reduced levels of L1 protein and E4 transcript in the carcinomas compared to dysplasia. This is consistent with our previous findings in the MmuPV1-induced cervicovaginal cancer model (28), in which we also observed reduced levels of L1 protein and E4 transcripts in virally induced cervical/vaginal cancers compared to precancerous lesions. Together, these results confirmed the presence of MmuPV1 in the oral tumors arising in the infected mice.

In addition to primary infection sites and associated disease, we also observed lateral transmission occurring on the infected tongue. Due to limits in accessibility, the primary infection sites were in the middle of the dorsal surface of the tongue. We frequently observed lateral transmission to the base of the tongue (Fig. S2A). The detection of MmuPV1 L1 confirmed the presence of virus at these secondary infection sites (Fig. S2B). This is consistent with a previous report that MmuPV1 preferentially targets the base of the tongue upon secondary, lateral infections (31).

10.1128/mBio.00908-20.2 FIG S2 Lateral transmission at the base of the MmuPV1-infected tongue. (A) Low-magnification scan of an H&E-stained, MmuPV1-infected tongue from a nude mouse. Primary infection and lateral transmission sites are identified by rectangles. (B, left) Higher-magnification images of the primary infection and lateral transmission sites stained with H&E. (Right) Both sites were positive for the viral capsid protein L1 (red signal, L1; green signal, K14; blue, DAPI). Download FIG S2, PDF file, 0.7 MB. Copyright © 2020 Wei et al. 2020 Wei et al. This content is distributed under the terms of the Creative Commons Attribution 4.0 International license.

### Biomarker analysis of MmuPV1-induced oral tumors arising in *Foxn1^nu/nu^* immunodeficient mice.

**FIG 4:**
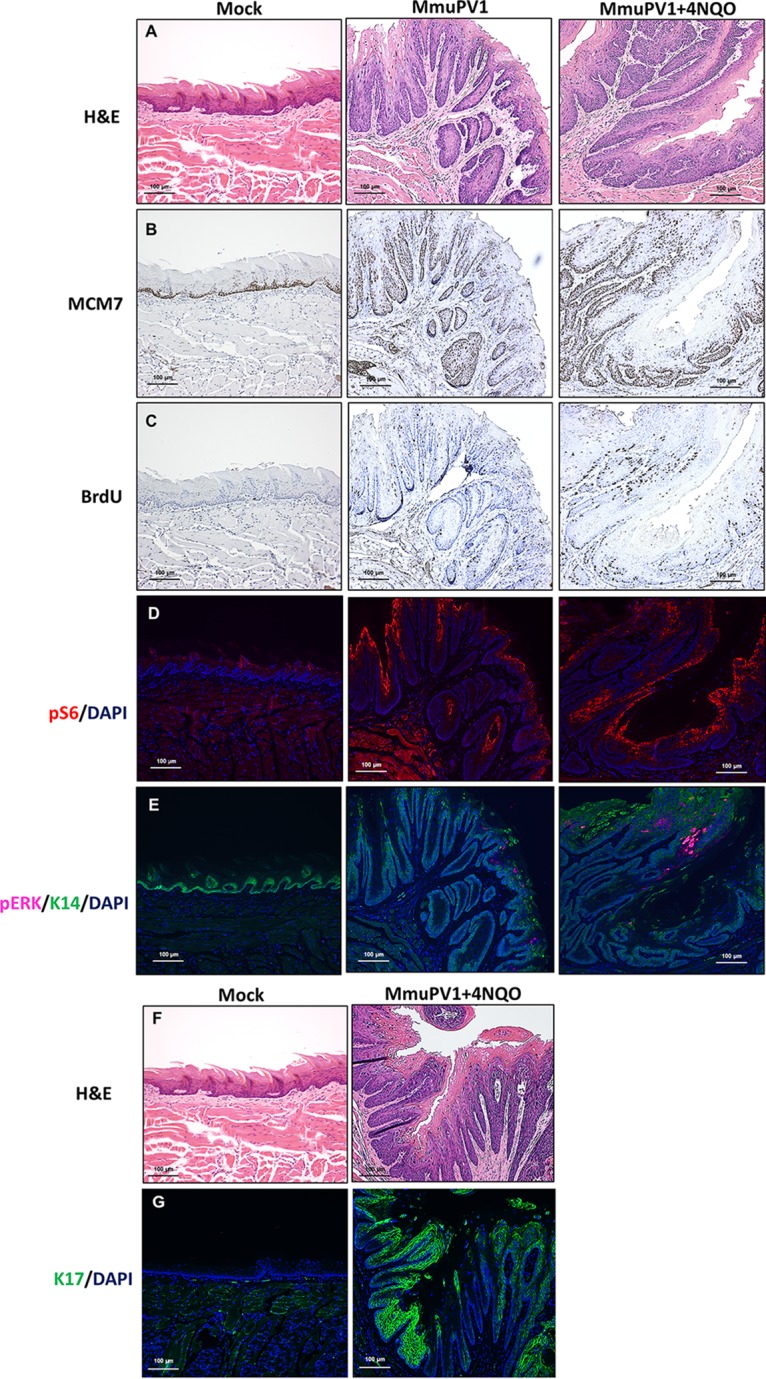
Biomarker analysis of MmuPV1-induced oral tumors arising in *Foxn1^nu/nu^* immunodeficient mice. Immunohistochemistry-based detection of MCM7 and BrdU was performed on groups of mock-infected, MmuPV1-infected, and MmuPV1-infected and 4NQO-treated mice. pERK was detected by TSA-enhanced immunofluorescence. Immunofluorescence detection of pS6 and keratin 17 was also performed on these experimental groups.

We assessed MmuPV1-induced oral tumors (Fig. 4A) with a panel of biomarkers. Levels of MCM7 protein, an E2F-responsive protein, were upregulated and expanded to the suprabasal compartment of the tongue epithelium in the MmuPV1-induced lesions while being restricted to the basal layers in the mock-infected epithelium, indicating an increased level of E2F transcription in the infected region (Fig. 4B). This feature is commonly observed in HPV-infected tissue and epithelia of HPV16 transgenic mice because of the inactivation of retinoblastoma (Rb) by the viral protein E7 (14, 28). Another feature found in HPV-induced lesions is an increased frequency of cells supporting DNA synthesis (14, 28), which we scored by measuring the incorporation of bromodeoxyuridine (BrdU), a nucleoside analog, in tissues from infected versus mock-infected mice. We observed increased numbers of BrdU-positive cells in the infected epithelium overall as well as increased numbers of BrdU-positive cells in the suprabasal compartment. Low levels of positive cells were present in the mock-infected epithelium, with no positive cells being observed in the suprabasal compartment (Fig. 4C).

We also tested two additional biomarkers of HPV-positive cancers. Phospho-S6 (pS6), the active form of the effector S6 for mTOR signaling, is upregulated in HPV-associated anal cancers (46, 47). We observed a similar upregulation of pS6 in our MmuPV1-induced tongue tumors (Fig. 4D). Another biomarker that is upregulated in HPV-positive cancers is phospho-extracellular signal-regulated kinase (pERK), the active form of the effector ERK for mitogen-activated protein kinase (MAPK) signaling (48, 49). Levels of pERK were elevated in our MmuPV1-induced tongue tumors, compared to those in mock-infected tongues (Fig. 4E). These results demonstrate that the tongue lesions caused by MmuPV1 infection resemble those associated with HPV infections, further validating the relevance of the use of MmuPV1 as an infection model for studying HPV-associated HNSCC.

One of the advantages of this infection model is the existence of normal-to-disease transition zones, which was also previously reported in our MmuPV1-induced cervical cancer paper (28). Changes in biomarker expression in the MmuPV1-infected regions were clearly observed when monitoring these transition zones. In both NSG and nude mice, levels of MCM7, BrdU, pERK, and pS6 were strongly upregulated in the L1-positive areas compared to the adjacent normal (uninfected, L1-negative) areas (Fig. S3).

10.1128/mBio.00908-20.3 FIG S3 Biomarker analysis of MmuPV1-induced disease transition junctions in the tongues of nude mice. The indicated sets of biomarker analyses were performed on oral tissues at disease transition junctions. Black/white arrows indicate the junction between normal and MmuPV1-infected areas. Download FIG S3, PDF file, 0.5 MB. Copyright © 2020 Wei et al. 2020 Wei et al. This content is distributed under the terms of the Creative Commons Attribution 4.0 International license.

Our laboratory recently discovered that stress cytokeratin 17 is one of the most upregulated host genes upon cutaneous MmuPV1 infection and that K17 plays an important role in mediating the ability of the virus to evade immune clearance and establish persistent disease (26). We observed that K17 is also upregulated in the context of MmuPV1-infected tongue lesions (Fig. 4F and G). The same observation was clearly found in the normal-to-disease transition zones in both NSG and nude mice, in which levels of K17 increased in the area positive for the presence of virus (Fig. S3). This indicates that K17 may be playing a similar role in preventing immune clearance of persistent, MmuPV1-induced lesions in the head and neck region.

### MmuPV1 infects and causes neoplastic lesions on the tongue of immunocompetent mice.

**FIG 5:**
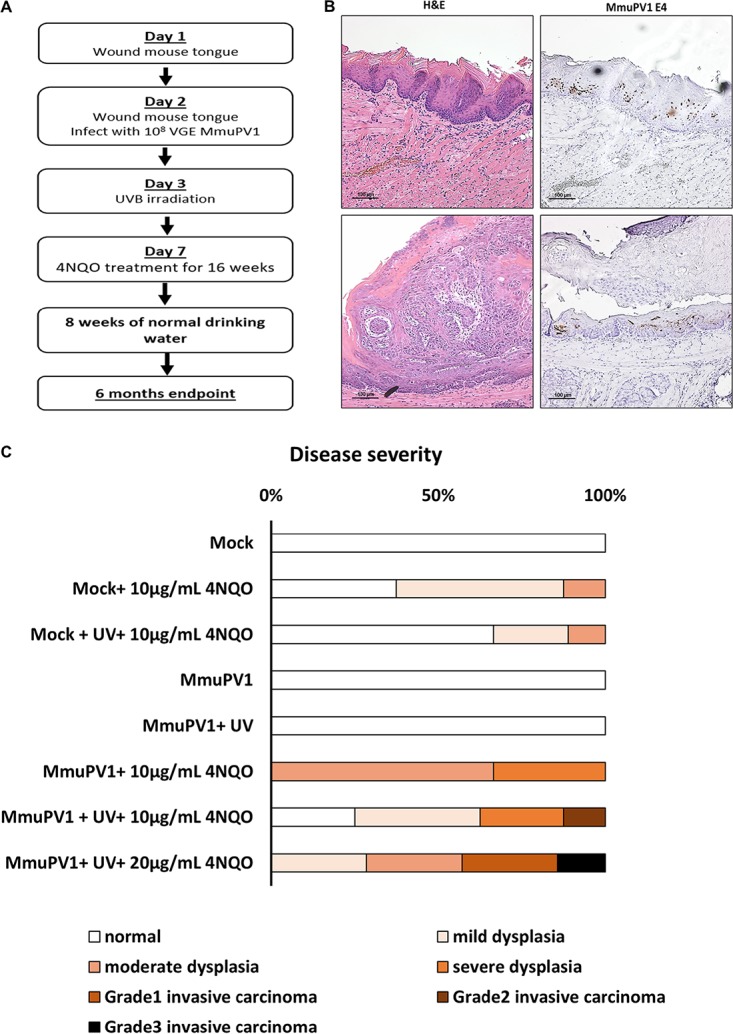
MmuPV1 infects and causes neoplastic lesions on the tongue of immunocompetent *FVB* mice, along with treatment with UVB and 4NQO. (A) Method for tongue infections in immunocompetent *FVB* mice with treatment with UVB irradiation and 4NQO. (B) MmuPV1-infected mice that were treated with both UVB and 20 μg/ml 4NQO showed signs of infection within neoplastic lesions. (C) Disease severity in each experimental group. Based on the *in situ* hybridization results, mice that were virus exposed but did not show signs of infection at the endpoint were excluded. Statistical analysis was performed with the two-sided Wilcoxon rank sum test. Detailed information on each experimental group is summarized in Table 2 and Table S1 in the supplemental material (score for mock versus MmuPV1, 0 versus 0 [*P* = 1]; score for mock plus 10 μg/ml 4NQO versus MmuPV1 plus 10 μg/ml 4NQO, 0.7 versus 2.3 [*P* = 0.02]; score for mock plus UV and 10 μg/ml 4NQO versus MmuPV1 plus UV and 10 μg/ml 4NQO, 0.4 versus 1.75 [*P* = 0.07]; score for mock plus UV and 10 μg/ml 4NQO versus MmuPV1 plus UV and 20 μg/ml 4NQO, 0.4 versus 2.8 [*P* = 0.002]; score for MmuPV1 versus MmuPV1 plus UV, 0 versus 0 [*P* = 1]; score for MmuPV1 versus MmuPV1 plus 10 μg/ml 4NQO, 0 versus 2.3 [*P* = 0.05]; score for MmuPV1 versus MmuPV1 plus UV and 10 μg/ml 4NQO, 0 versus 1.75 [*P* = 0.06]; score for MmuPV1 versus MmuPV1 plus UV and 20 μg/ml 4NQO, 0 versus 2.8 [*P* = 0.01]).

We extended our study to establish an MmuPV1 infection model for HNSCC in genetically immunocompetent *FVB* mice. Previous work in our laboratory suggested the use of UVB as a cofactor to induce systematic immunosuppression, which increased susceptibility to MmuPV1 in genetically immunocompetent mice (24). We therefore incorporated the use of both UVB and 4NQO into our experimental design for studies on *FVB* mice (Fig. 5A). Mice were wounded and infected as described above. Twenty-four hours after infection, mice were or were not treated with 600 mJ/cm^2^ of UVB. Starting at 7 days postinfection, mice were or were not put on a 16-week-long 4NQO treatment with either 10- or 20-μg/ml doses in their drinking water, followed by 8 weeks of water without the carcinogen. The tissues were harvested and processed at week 25 postinfection.

10.1128/mBio.00908-20.7 TABLE S1 Disease severity of all experimental *FVB* mice in the study, with or without UV and 4NQO treatment, including those excluded from Table 2. Numbers in parentheses indicate the number of mice that were negative for the MmuPV1 E4 transcript by *in situ* hybridization, which were the samples that were excluded from Table 2. M, male; F, female. Download Table S1, PDF file, 0.1 MB. Copyright © 2020 Wei et al. 2020 Wei et al. This content is distributed under the terms of the Creative Commons Attribution 4.0 International license.

At the endpoint, we were able to detect the presence of viral mRNAs by *in situ* hybridization against MmuPV1 E4 transcripts in all infected groups (Fig. 5B and Fig. S4), indicating that we successfully infected this genetically immunocompetent strain. Most of these persistent infections remained asymptomatic, represented by the relatively normal appearance of the squamous epithelium or only mild dysplasia in the corresponding H&E images (Fig. 5B, top). However, we also observed viral signals in tumors arising in some of the MmuPV1-infected mice (Fig. 5B, bottom). UVB and 4NQO, alone or together, did not significantly change the incidence of persistent infections on the mouse tongues (Fig. S5), but they affected the severity of disease (Fig. 5C). Based on the pathological evaluation, MmuPV1 virus alone as well as combined with the UVB cofactor did not cause any neoplastic disease at the 6-month endpoint. However, 4NQO treatment significantly increased disease severity in MmuPV1-infected mice (mean score for “MmuPV1” versus “MmuPV1 plus 10 μg/ml 4NQO,” 0 versus 2.3 [*P* {two sided} = 0.05]). The combination of both UVB and 4NQO also promoted greater disease severity, especially at the higher dosage of 4NQO (mean score for MmuPV1 versus MmuPV1 plus UV and 10 μg/ml 4NQO, 0 versus 1.75 [*P* {two sided} = 0.06]; mean score for MmuPV1 versus MmuPV1 plus UV and 20 μg/ml 4NQO, 0 versus 2.8 [*P* {two sided} = 0.01]). This indicated that the cofactor 4NQO primarily contributes to disease progression in this infection-based model for HNSCC.

10.1128/mBio.00908-20.4 FIG S4 MmuPV1 E4 transcripts detected by *in situ* hybridization at sites of infection on the tongues of *FVB* mice. Many of these infections were asymptomatic. Download FIG S4, PDF file, 0.6 MB. Copyright © 2020 Wei et al. 2020 Wei et al. This content is distributed under the terms of the Creative Commons Attribution 4.0 International license.

10.1128/mBio.00908-20.5 FIG S5 Incidence of infection in each group of MmuPV1-infected *FVB* mice (*P* = 1 for “MmuPV1 only” versus “MmuPV1 plus UV”; *P* = 1 for MmuPV1 only versus MmuPV1 plus 10 μg/ml 4NQO; *P* = 0.23 for MmuPV1 only versus MmuPV1 plus UV and 10 μg/ml 4NQO; *P* = 1 for MmuPV1 only versus MmuPV1 plus UV and 10 μg/ml 4NQO [by two-sided Fisher’s exact test]). Download FIG S5, PDF file, 0.2 MB. Copyright © 2020 Wei et al. 2020 Wei et al. This content is distributed under the terms of the Creative Commons Attribution 4.0 International license.

MmuPV1 clearly influenced disease development (Fig. 5C). In the presence of 4NQO, MmuPV1 significantly increased disease severity in the infected group compared to the mock-infected 4NQO-treated group (mean score for mock plus 10 μg/ml 4NQO versus MmuPV1 plus 10 μg/ml 4NQO, 0.7 versus 2.3 [*P* {two sided} = 0.02]). We noticed the same effect when the mice were treated with both UVB and 4NQO, especially with the higher dosage of 4NQO, in which infection with MmuPV1 was associated with higher overall disease severity (mean score for mock plus UV and 10 μg/ml 4NQO versus MmuPV1 plus UV and 10 μg/ml 4NQO, 0.44 versus 1.75 [*P* {two sided} = 0.07]; mean score for mock plus UV and 10 μg/ml 4NQO versus MmuPV1 plus UV and 20 μg/ml 4NQO, 0.44 versus 2.8 [*P* {two sided} = 0.002]). Detailed histopathological scoring results are summarized in Table 2 and Table S1. Together, our results indicate that MmuPV1 infection contributes to the severity of disease progression on the tongue of genetically immunocompetent mice.

**TABLE 2:** Disease severity in MmuPV1-infected FVB mice, with or without UV and 4NQO treatment^*a*^

Group	Total no. of mice	No. of mice of gender	No. of mice with disease severity						
			Normal	Dysplasia			Invasive carcinoma		
				Mild	Moderate	Severe	Grade 1	Grade 2	Grade 3
Mock	5	2 M, 3 F	5	0	0	0	0	0	0
Mock + 10 μg/ml 4NQO	8	8 M	3	4	1	0	0	0	0
Mock + UV + 10 μg/ml 4NQO	9	5 M, 4 F	6	2	1	0	0	0	0
MmuPV1	3	2 M, 1 F	3	0	0	0	0	0	0
MmuPV1 + UVB	3	2 M, 1 F	3	0	0	0	0	0	0
MmuPV1 + 10 μg/ml 4NQO	3	3 F	0	0	2	1	0	0	0
MmuPV1 + UV + 10 μg/ml 4NQO	8	4 M, 4 F	2	3	0	2	0	1	0
MmuPV1 + UV + 20 μg/ml 4NQO	7	6 M, 1 F	0	2	2	0	2	0	1

a Mice that were infected with MmuPV1 but did not show signs of infection at the endpoint were excluded. Infection status was determined by *in situ* hybridization. See Table S1 in the supplemental material for the inclusion of data from mice that were infected with MmuPV1 but did not show signs of infection at the endpoint. M, male; F, female.

In addition, we also observed a significant difference in disease severity upon MmuPV1 infection based on sex. Among the mock-infected mice treated with 4NQO and UVB, there was no difference in disease severity between male and female mice (mean score for male versus female mice, 0.4 versus 0.5 [*P* {two sided} = 1]). Among the MmuPV1-infected mice treated with both 4NQO and UVB, male mice developed significantly more severe disease than did female mice (mean score for male versus female mice, 2.6 versus 0.5 [*P* {two sided} = 0.049]). This result is consistent with the observation that male patients are more implicated in human HPV-positive HNSCCs (50).

We performed the same biomarker analyses on lesions arising in *FVB* mice (Fig. S6) as in the immunodeficient mice. The lesions from the MmuPV1-infected mice treated with both UVB and 20 μg/ml 4NQO showed upregulated levels of MCM7 and BrdU, the two common markers in HPV-infected lesions. The levels of the two markers for HPV-associated cancer, pS6 and pERK, were also increased in the MmuPV1-induced lesions. These results together suggested that the oral tumors arising in MmuPV1-infected genetically immunocompetent *FVB* mice also resemble HPV-associated tumors. In addition, we again observed high levels of K17 in the MmuPV1-induced lesions, indicating the possible role played by K17 in evading the immune clearance of MmuPV1 infection, similar to that in cutaneous infection.

10.1128/mBio.00908-20.6 FIG S6 Biomarker analysis of MmuPV1-induced oral tumors in *FVB* immunocompetent mice. Representative H&E-stained images of mock-infected and MmuPV1-infected mice treated with UV and 20 μg/ml 4NQO are shown in the top panels. Immunohistochemistry detection of MCM7 and BrdU was performed between mock-infected and MmuPV1-infected plus 4NQO-treated mice. The capsid protein L1 and pERK were detected by immunofluorescence with TSA treatment. Immunofluorescence detection of pS6 and keratin 17 was also performed on these two experimental groups. Download FIG S6, PDF file, 0.6 MB. Copyright © 2020 Wei et al. 2020 Wei et al. This content is distributed under the terms of the Creative Commons Attribution 4.0 International license.

## DISCUSSION

In this study, we developed an *in vivo* infection model for studying papillomavirus-induced HNSCCs using MmuPV1. First, we showed that using our infection method, MmuPV1 could infect and cause high-grade dysplasia in immunodeficient mouse strains by 16 weeks. With the addition of the oral carcinogen 4NQO, invasive SCC was induced in MmuPV1-infected nude mice. The MmuPV1-induced tumors harbored features associated with papillomavirus infection (increased levels of MCM7, BrdU, and K17) as well as HPV-related carcinogenesis (increased levels of pS6 and pERK). In genetically immunocompetent *FVB* mice, we were able to infect the squamous tongue epithelium with MmuPV1, although many of these infections were asymptomatic, without grossly visible lesions. Treatment with cofactors, both UVB and 4NQO or 4NQO alone, promoted more severe neoplastic disease in MmuPV1-infected mice, with infection being associated with higher disease severity. The tumors arising in the MmuPV1-infected *FVB* mice expressed similar biomarkers as those seen in HPV-associated HNSCC in patients.

Cladel et al. (31) previously showed that MmuPV1 can infect the tongue epithelium of nude mice and cause mild disease by 8 months postinfection. Here, we describe all stages of HNSCC progression upon MmuPV1 infection. Invasive SCC arose in MmuPV1-infected nude mice upon 4NQO treatment by 6 months postinfection (Fig. 2D and Table 1). Likewise, MmuPV1 infection of genetically immunocompetent mice induced squamous dysplasia and, with the addition of 4NQO alone or in combination with UVB, caused invasive SCC (Fig. 5C). Viral infections in nude mice induced much higher-grade disease, and the tumor sites usually contained stronger signals for MmuPV1 L1 (Fig. 3). In contrast, in *FVB* mice, a large portion of infections remained asymptomatic (Fig. 5B; see also Fig. S4 in the supplemental material). These infection sites also expressed much lower levels of L1, often undetectable (data not shown), indicating a paucity in the productive phase of the viral life cycle in *FVB* mice. This is consistent with our observations of cutaneous MmuPV1 infections in which warts from nude mice expressed significantly stronger L1 signals than those arising in *FVB* mice (24). It is likely that the immune system is playing a large role in this difference, which is supported by the lack of disease caused by MmuPV1 infection alone in *FVB* mice at the 6-month endpoint (Fig. 5C), while high-grade dysplasia developed within 4 months in nude mice that lack functional T cells (Fig. 2D). However, we cannot rule out the possibility that those samples that tested negative for MmuPV1 E4 transcripts at the endpoint might still be infected, considering that some HPV-positive HNSCCs display high levels of p16, a clinically used marker for determining HPV infection, but show very low to nondetectable levels of viral E6 and E7 transcripts (51).

In our study, the addition of 4NQO advanced the development of MmuPV1-induced invasive carcinoma in nude mice (Fig. 2D and Table 1) and *FVB* mice, in which carcinoma occurred only when mice were treated with 4NQO (Fig. 5C). Our data indicate that 4NQO did not affect the ability of the virus to establish persistent infection *per se* (Fig. S5); rather, it contributed to disease by driving malignant progression. 4NQO is a synthetic oral carcinogen that causes DNA damage similar to that caused by tobacco-associated carcinogens (35). Exome sequencing also showed that cell lines derived from 4NQO-induced murine oral cancer mimic human tobacco-related human HNSCCs and exhibit typical histology and mutations found in human HNSCCs (36). Tobacco use in HPV-positive HNSCC patients is associated with more aggressive disease progression and lower survival rates (52–54). Previous work has shown 4NQO to be a potent cofactor in driving HPV-associated HNSCC in HPV16 transgenic mice (14). 4NQO-induced carcinogenesis is driven, at least in part, by the overexpression of epidermal growth factor receptor (EGFR) (35), which is upstream of the phosphatidylinositol 3-kinase (PI3K) signaling pathway, the most altered signaling pathway in HPV-positive HNSCCs (55–57). The PI3K signaling pathway mediates multiple cellular processes, including cell survival, migration, and proliferation, and it is commonly implicated in cancer development (57, 58). Our data demonstrated that lesions induced by MmuPV1 infection showed an upregulation of PI3K signaling (Fig. 4, Fig. S3, and Fig. S6), indicating that this pathway may be important in MmuPV1-induced disease, as is thought to be the case in HPV-induced disease. Another possibility is that it activates the Ras-MAPK pathway, which is also downstream of EGFR and is also implicated in cancer development (59). Our data show that the Ras-MAPK pathway was also upregulated in MmuPV1-induced lesions (Fig. 4, Fig. S3, and Fig. S6). Future studies will be needed to understand the mechanism of how 4NQO contributes to MmuPV1-induced neoplastic disease.

The cofactor UVB increases the susceptibility of immunocompetent mice to MmuPV1 infection in the cutaneous epithelium by inducing systemic immunosuppression (24, 60). However, in our study, UVB did not have a similar effect on the head and neck region. UVB treatment did not affect the incidence of persistent infections in *FVB* mice (Fig. S5). UVB, either alone or combined with 4NQO, did not impact disease severity in MmuPV1-infected *FVB* mice either (mean score for MmuPV1 versus MmuPV1 plus UV, 0 versus 0 [*P* = 1]; mean score for MmuPV1 plus 10 μg/ml 4NQO versus MmuPV1 plus UV and 10 μg/ml 4NQO, 2.3 versus 1.75 [*P* = 0.49]). This could be due to the intrinsic differences between cutaneous and mucosal epithelia (61, 62), making it possible that the two sites react to UVB differently. Consistent with this hypothesis, our laboratory’s previous study also showed that in the female reproductive tract, UVB treatment alone did not significantly increase disease severity in MmuPV1-infected *FVB* mice (28). It is not clear at this point whether or how the roles of UVB in MmuPV1-induced disease are different between mucosal and cutaneous sites.

In the MmuPV1 cervicovaginal infection model, MmuPV1 alone caused cancer in *FVB* mice at 6 months postinfection (28). However, in our oral infection model, MmuPV1 infection alone failed to cause any disease on the tongues of *FVB* mice by the end of 6 months (Fig. 5C and Table 2). This difference could have multiple explanations. The microenvironments of the cervix and oral squamous mucosa are very different (63, 64), and genome-wide gene expression profiling has shown that HPV-positive HNSCCs differ in their gene expression patterns from HPV-positive cervical cancers (64). It is also possible that viral infections in different organs differ in their intensity. A previous study that longitudinally tracked MmuPV1 infections in different organs reported that in the oral cavity, a detectable presence of the virus appeared later than in the cervix and anus and that the viral genome was present at a lower copy number in the oral cavity (32). The virus also could have a different oncogenic potential in the oral cavity than in the female reproductive tract. This is supported by our data in nude mice, where MmuPV1 infection alone caused high-grade dysplasia only in the oral cavity (Fig. 2D) but resulted in invasive carcinoma of the cervix and vagina (28). This difference may be a consequence of the differences in the intensities of the infections (including viral copy numbers per cell and numbers of infected cells, etc.) in the two organs and/or due to differences in the relevance of virally encoded oncogenic activities in the process of carcinogenesis. Another factor contributing to organ differences could be the immune system. The oral cavity has very a different immunological environment than the cervix. Immune marker profiling has demonstrated that the oral cavity has a significantly higher concentration of T cell-related immune markers than the cervix, including higher levels of interleukin-2 (IL-2), an important T cell response marker that is often reduced during cervical HPV infection (63). Because T cell responses play crucial roles in MmuPV1 infection and associated disease (23, 24, 26, 65), the oral cavity may be better protected against viral infection and associated tumorigenesis than the female reproductive tract because of the increased immunological surveillance in the former. This would support the hypothesis that MmuPV1 causes less severe disease in the tongue than in the cervix/vagina because it is more difficult for the virus to establish, maintain, and mediate pathogenesis in the oral cavity.

To summarize, we present an *in vivo* infection model for studying papillomavirus-associated HNSCC using MmuPV1. By combining the use of an oral carcinogen with MmuPV1 infection, we were able to induce MmuPV1-associated invasive carcinoma of the mouse tongue epithelium for the first time. This opens the door to answering many pressing questions in the papillomavirus field, such as the establishment of persistent infections by papillomavirus and viral manipulation of the host immune system. With the MmuPV1-induced HNSCC model in genetically immunocompetent mice, we also can develop and test the efficacy of immunomodulatory drugs or therapeutic vaccines for papillomavirus-mediated head and neck disease.

## MATERIALS AND METHODS

### Mice.

This study included the use of immunodeficient nude mice (Hsd:Athymic Nude-*Foxn1^nu^*, all females; Envigo) and immunocompetent *FVB/N* mice (Taconic). Immunodeficient NSG mice (stock number 005557; Jackson Laboratory) were bred by the UW-Madison Biomedical Research Models Services Laboratory. All mice were housed in the UW School of Medicine and Public Health (SMPH) Animal Care Unit approved by the Association for Assessment and Accreditation of Laboratory Animal Care. All procedures were carried out in accordance with an animal protocol approved by the UW SMPH Institutional Animal Care and Use Committee.

### MmuPV1 infection of the tongue epithelium.

An MmuPV1 virus stock was generated by isolating virions from papillomas in nude mice as described previously (24). The MmuPV1 tongue epithelium infection model was adapted from methods reported previously (31). Briefly, mice were put under isoflurane-induced anesthesia until the surgical tolerance stage. The mice were then removed from the anesthesia-inducing chamber. Quickly, the tongue was drawn out using flat-top forceps and lightly wounded on the dorsal surface by using either 18-gauge syringe needles (nude mouse study) or Greer Pick (Greer Laboratories, Inc., Lenoir, NC) (*FVB* mouse study). The utilization of the Greer Pick for MmuPV1 infection is described in detail elsewhere (68). Twenty-four hours later, the mice were wounded lightly in the same way, and either phosphate-buffered saline (PBS) only (mock infection) or 10^8^ viral genome equivalence (VGE) of MmuPV1 in PBS were then pipetted onto the wounded site. Over the experimental period, mice were checked monthly or at other indicated time points for overt tumor formation.

### UVB radiation.

The indicated groups of *FVB* mice were exposed to a single dose of UVB at 600 mJ/cm^2^ as described previously (24, 60), at 24 h postinfection. UVB irradiation was delivered using a custom-designed research irradiation unit (Daavlin, Bryan, OH).

### 4NQO-induced HNSCC treatment.

For the nude mouse study, mice were treated with 4NQO (Sigma) in their drinking water at 10 μg/ml (stored at 4°C; 1-mg/ml stock) for 8 weeks and then returned to normal drinking water for 16 weeks. For the *FVB* mouse study, the mice were treated with 10 or 20 μg/ml 4NQO for 16 weeks, followed by 8 weeks on normal drinking water.

### Overt tumor and histological analyses.

At the endpoint, mice were euthanized, and the numbers of grossly visible, overt tumors on the tongue were quantified. The tissues were then collected, fixed in 4% paraformaldehyde for 24 h, switched to 70% ethanol for 24 h, processed, paraffin embedded, and sectioned at 5-μm intervals. Every 10th section was stained with H&E and examined by a pathologist, D. Buehler, in a blind fashion to assess the presence and severity of squamous dysplasia (mild, moderate, or severe/CIS) and invasive carcinoma (well differentiated [grade 1], moderately differentiated [grade 2], or poorly differentiated [grade 3]).

### Oral swabbing and detection of MmuPV1 by PCR.

The method for detecting the MmuPV1 E2 gene in oral swab samples by PCR was adapted and modified from previously reported work (29, 32). Briefly, tongues of anesthetized mice were drawn out by forceps, and the infection sites were swabbed multiple times using a flat toothpick. The portion of the toothpick exposed to the tongue was placed into 50 μl of sterile PBS and stored at −80°C. DNA from the swab samples was isolated using the DNeasy blood and tissue kit (catalog number 69506; Qiagen, Hilden, Germany). MmuPV1 E2 and p53 PCR conditions were described previously (28). Oral swab samples were collected from all mock/MmuPV1-infected mice at the indicated times.

### BrdU incorporation.

We measured bromodeoxyuridine (BrdU) incorporation to evaluate the levels of DNA synthesis. Mice were intraperitoneally injected with 250 μl of BrdU (Sigma) (12.5 mg/ml in PBS, stored at −20°C) 1 h before euthanasia. Tissues were harvested and processed for immunohistochemistry.

### Immunohistochemistry.

A representative slide was selected based on histology scoring, and at least three samples were included for each group. Tissue sections were deparaffinized in xylene and rehydrated in graded ethanol. Heat-induced antigen unmasking was performed in 10 mM citrate buffer (pH 6). Slides were blocked with 2.5% horse serum for 1 h at room temperature (RT) and then incubated with primary antibody at 4°C overnight in a humidified chamber. A M.O.M. ImmPRESS HRP (horseradish peroxidase) polymer kit (catalog number MP-2400; Vector) was applied the next day for 1 h at RT for secondary antibody incubation. Slides were then incubated with 3,3′-diaminobenzidine (Vector Laboratories) and counterstained with hematoxylin. All images were taken with a Zeiss AxioImager M2 microscope using AxioVision software version 4.8.2.

For immunofluorescence, tissue sections were deparaffinized, subjected to antigen unmasking as described above, and blocked in tyramide-based signal amplification (TSA) blocking buffer (catalog number FP1012; PerkinElmer). After overnight incubation with primary antibody, Alexa Fluor 488 or 594 goat anti-rabbit (Invitrogen) (1:500) was applied the next day as the secondary antibody. Sections were counterstained with DAPI (4′,6-diamidino-2-phenylindole) and mounted in Prolong diamond antifade mountant (Invitrogen). L1 and pERK signals were detected using a TSA method (66). A detailed protocol for TSA can be found at https://www.protocols.io/view/untitled-protocol-i8cchsw.

Information for all primary antibodies is summarized in Table S2 in the supplemental material.

10.1128/mBio.00908-20.8 TABLE S2 Summary of all primary antibodies used in this study. Download Table S2, PDF file, 0.1 MB. Copyright © 2020 Wei et al. 2020 Wei et al. This content is distributed under the terms of the Creative Commons Attribution 4.0 International license.

### RNA *in situ* hybridization.

Representative slides were selected for each MmuPV1-infected mouse sample based on histology scoring, along with 2 representative slides from each corresponding mock-infected group. *In situ* hybridization was performed using RNAscope 2.5 HD assay—brown (Advanced Cell Diagnostic, Newark, CA) according to the manufacturer’s instructions (67). Signals for the viral transcript were detected using MmuPV1 E4 probes (catalog number 473281; Advanced Cell Diagnostic).

### Statistical analysis.

For disease severity, each microscopic tumor grade was assigned a ranking order (1 for mild, 2 for moderate, and 3 for severe dysplasia and 4 for grade 1, 5 for grade 2, and 6 for grade 3 invasive carcinoma). A Wilcoxon rank-sum test was performed to determine the significance of differences in disease severity using MSTAT statistical software version 6.4.2 (http://www.mcardle.wisc.edu/mstat). Fisher’s exact test was performed to determine the significance of differences in infection incidences between experimental groups at the endpoint, using MSTAT.
