# Supplementary figures and images for: An Infection-Based Murine Model for Papillomavirus-Associated Head and Neck Cancer
Source: mBio. 2020 May 12;11(3):e00908-20. doi: 10.1128/mBio.00908-20 (PMC7218285; doi:10.1128/mBio.00908-20)

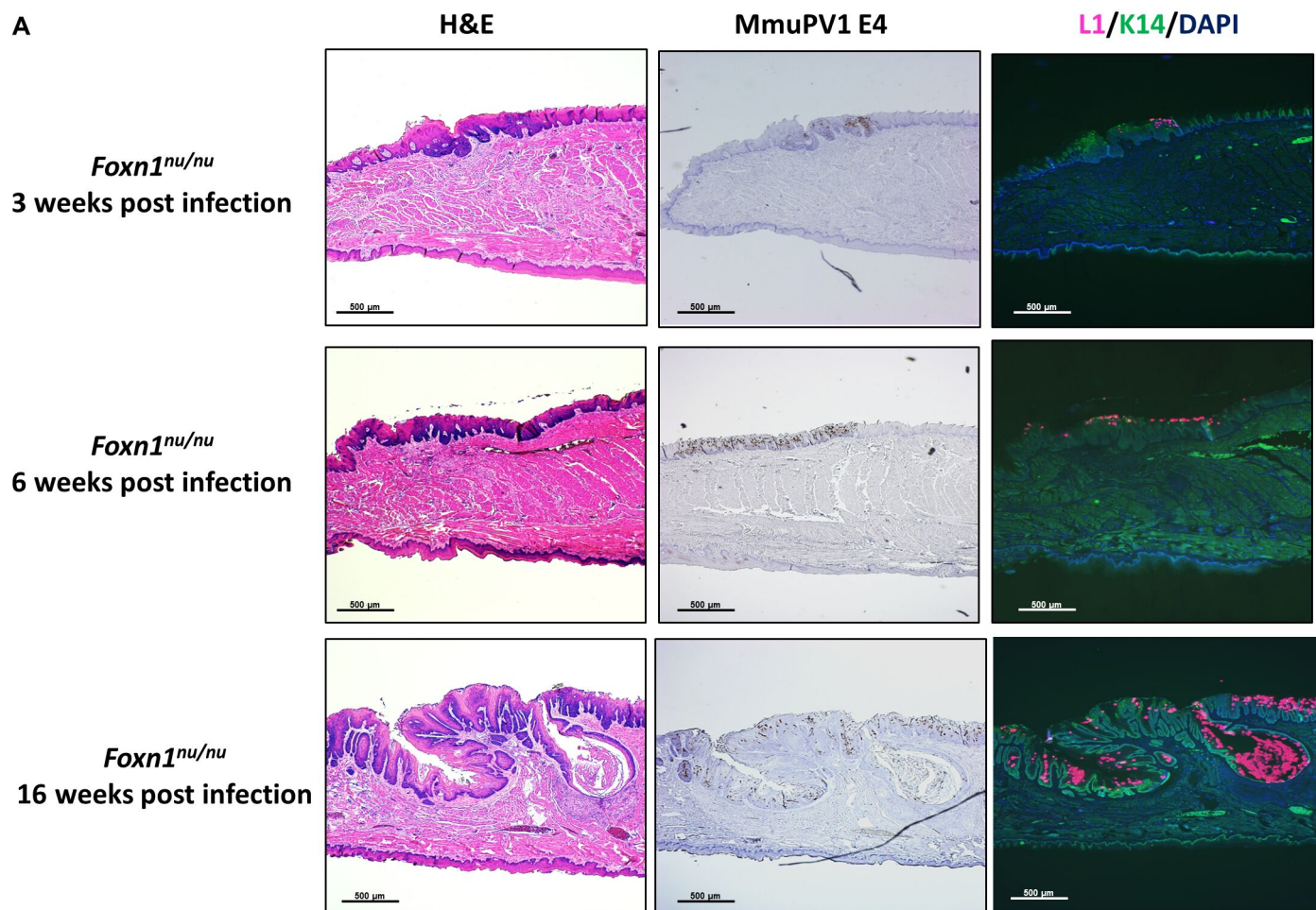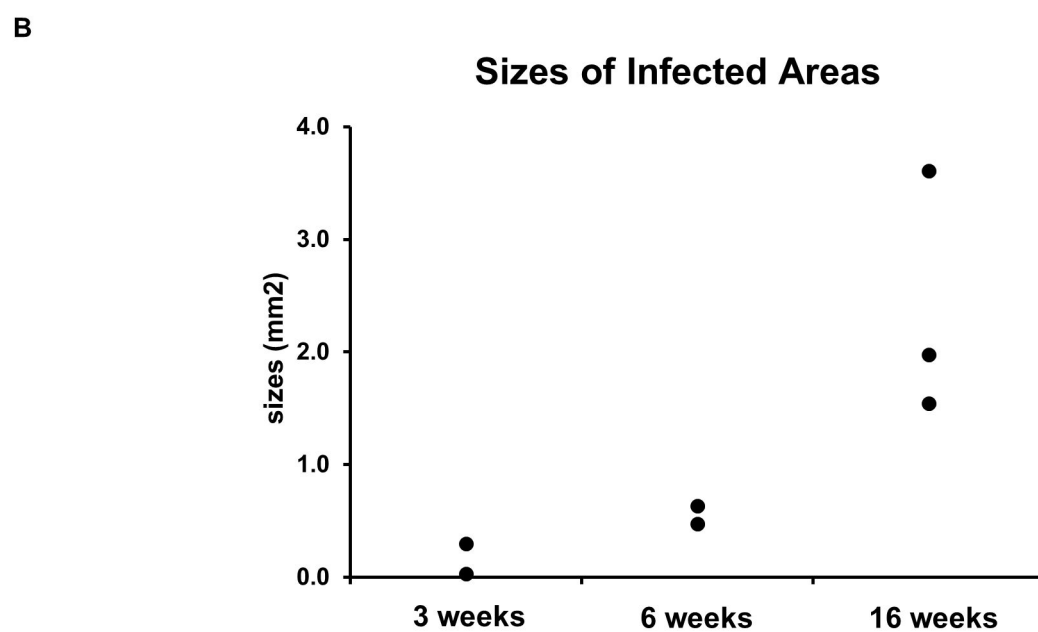

Supplement: FIG S1 [file mBio.00908-20-sf001.pdf]

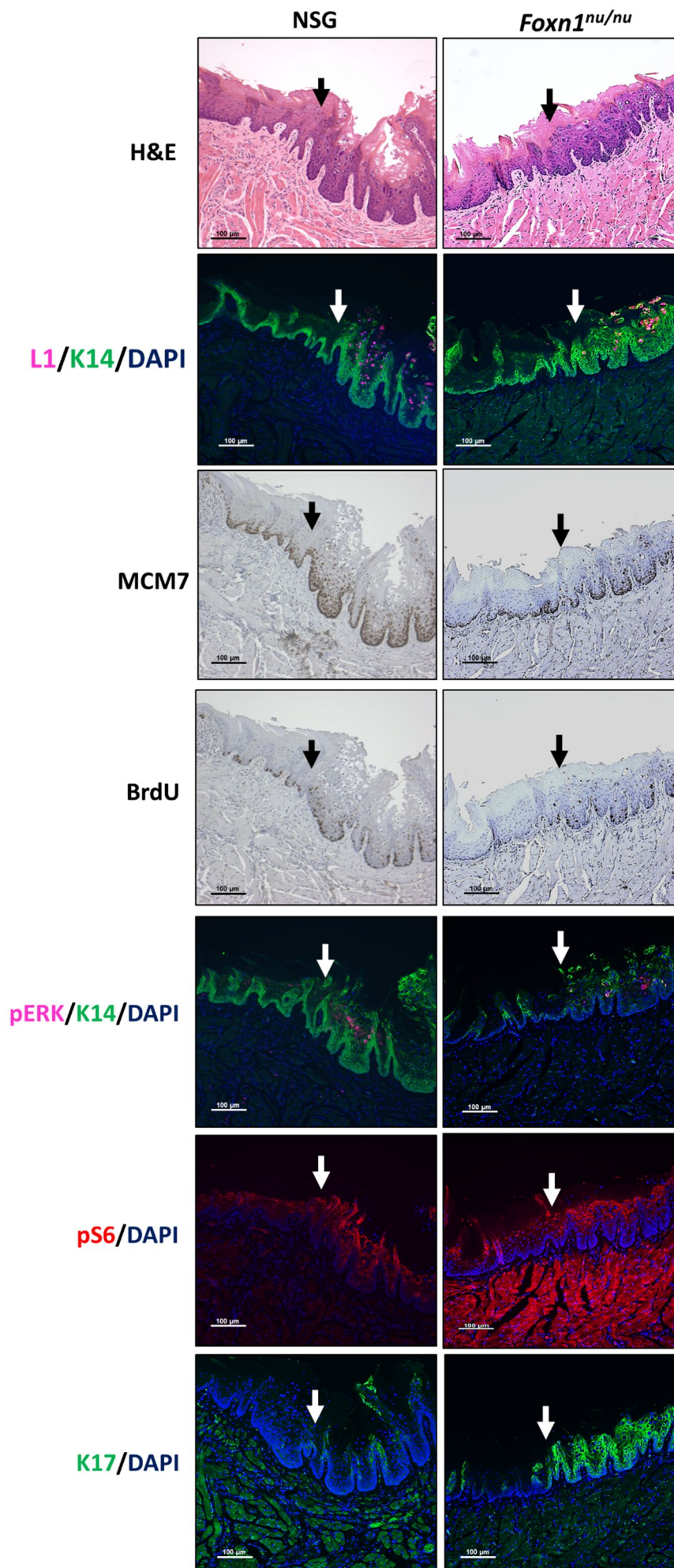

Supplement: FIG S3 [file mBio.00908-20-sf003.pdf]

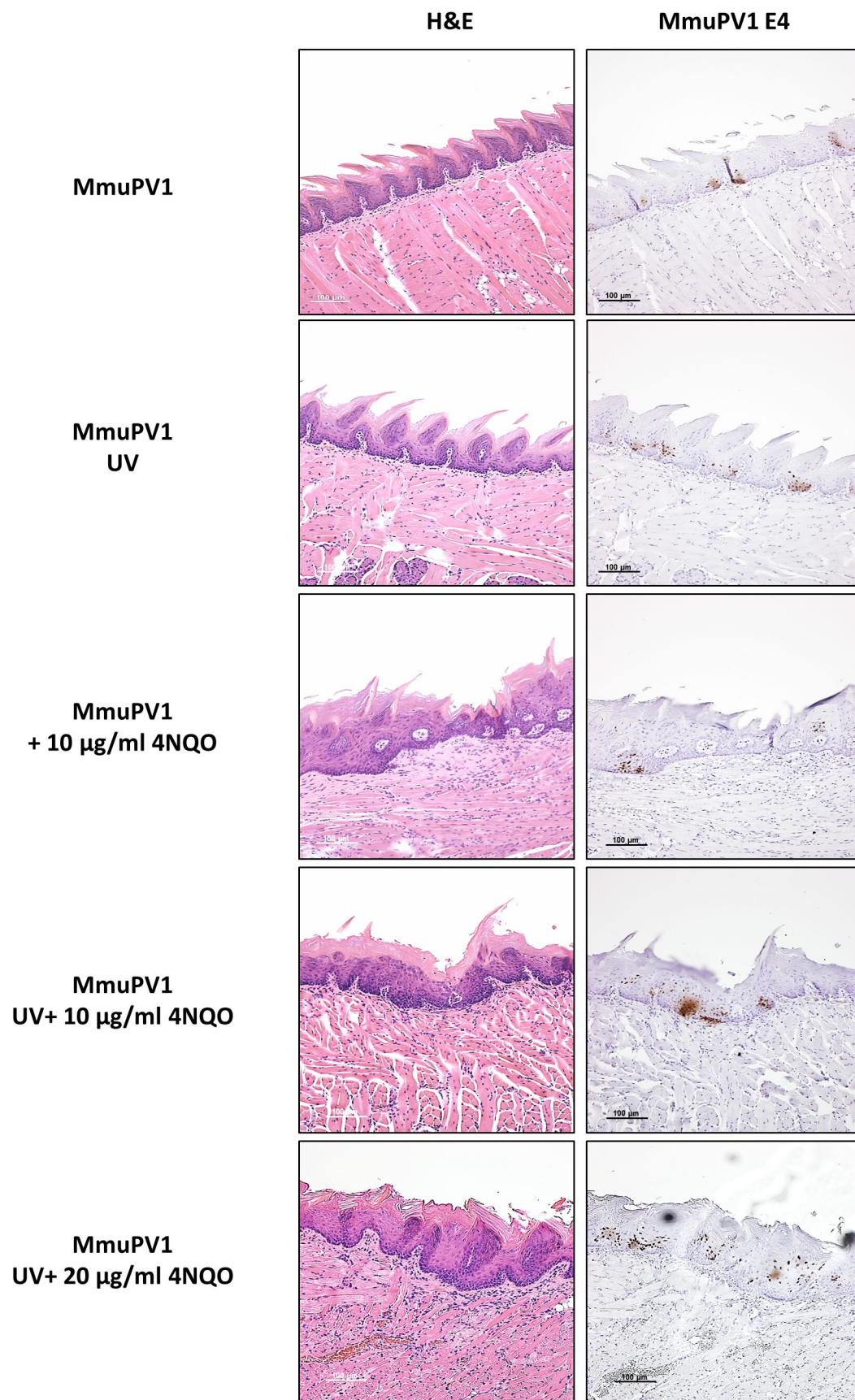

Supplement: FIG S4 [file mBio.00908-20-sf004.pdf]

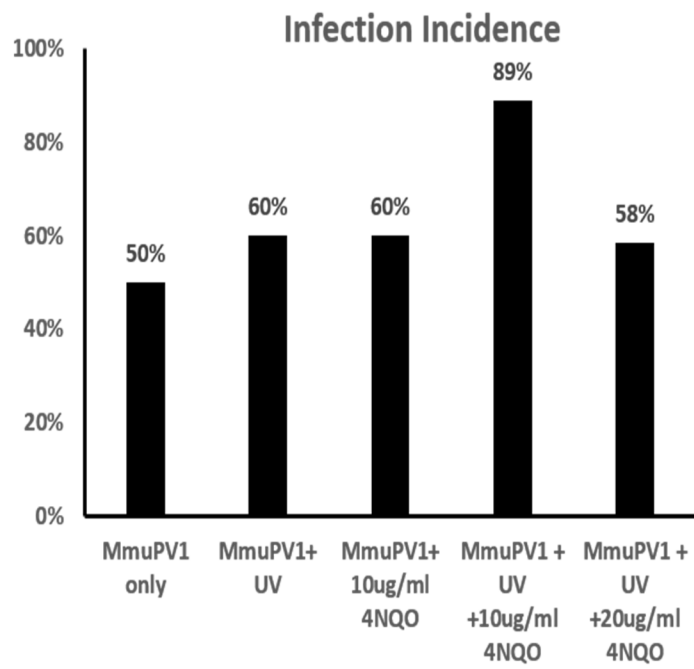

Supplement: FIG S5 [file mBio.00908-20-sf005.pdf]

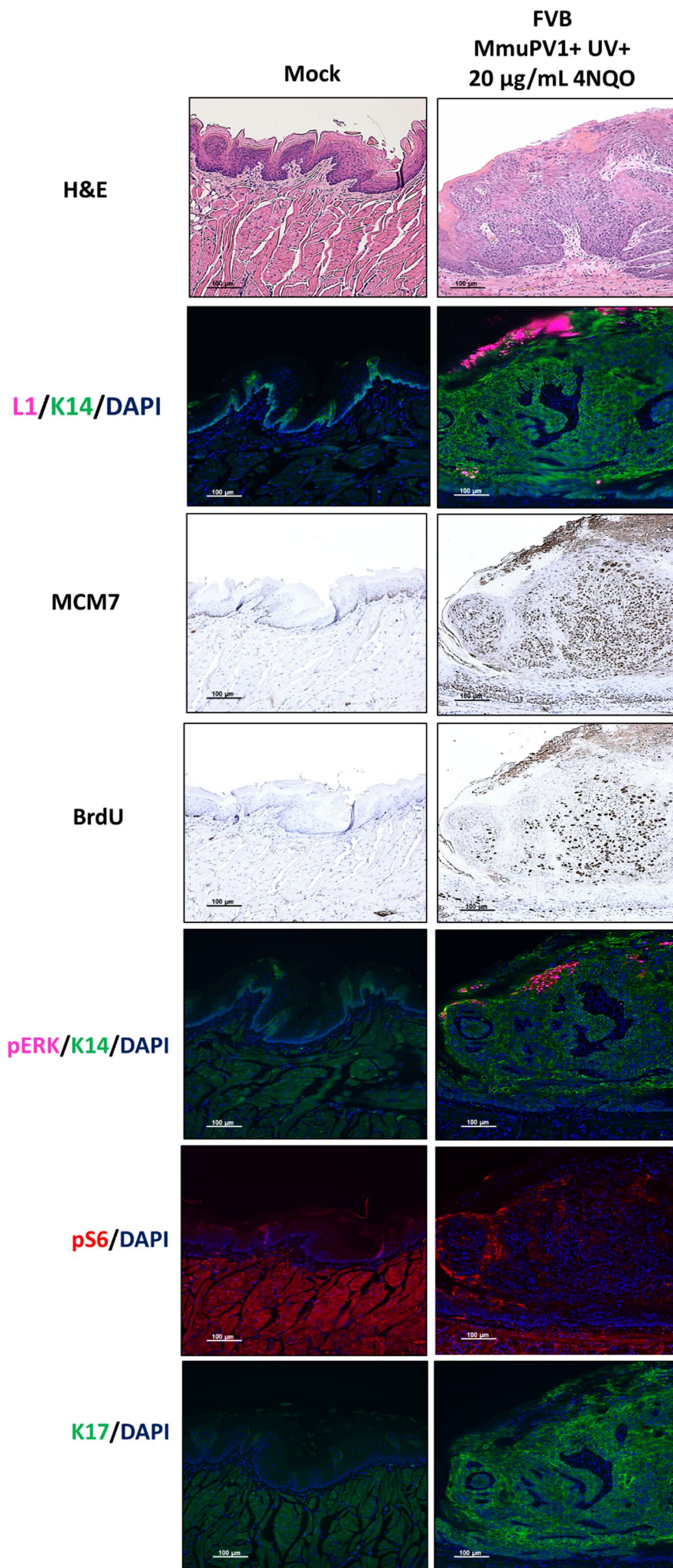

Supplement: FIG S6 [file mBio.00908-20-sf006.pdf]
