# Supplementary material for: An Infection-Based Murine Model for Papillomavirus-Associated Head and Neck Cancer
Source: mBio. 2020 May 12;11(3):e00908-20. doi: 10.1128/mBio.00908-20 (PMC7218285; doi:10.1128/mBio.00908-20)
Supplement: FIG S2 [file mBio.00908-20-sf002.pdf]

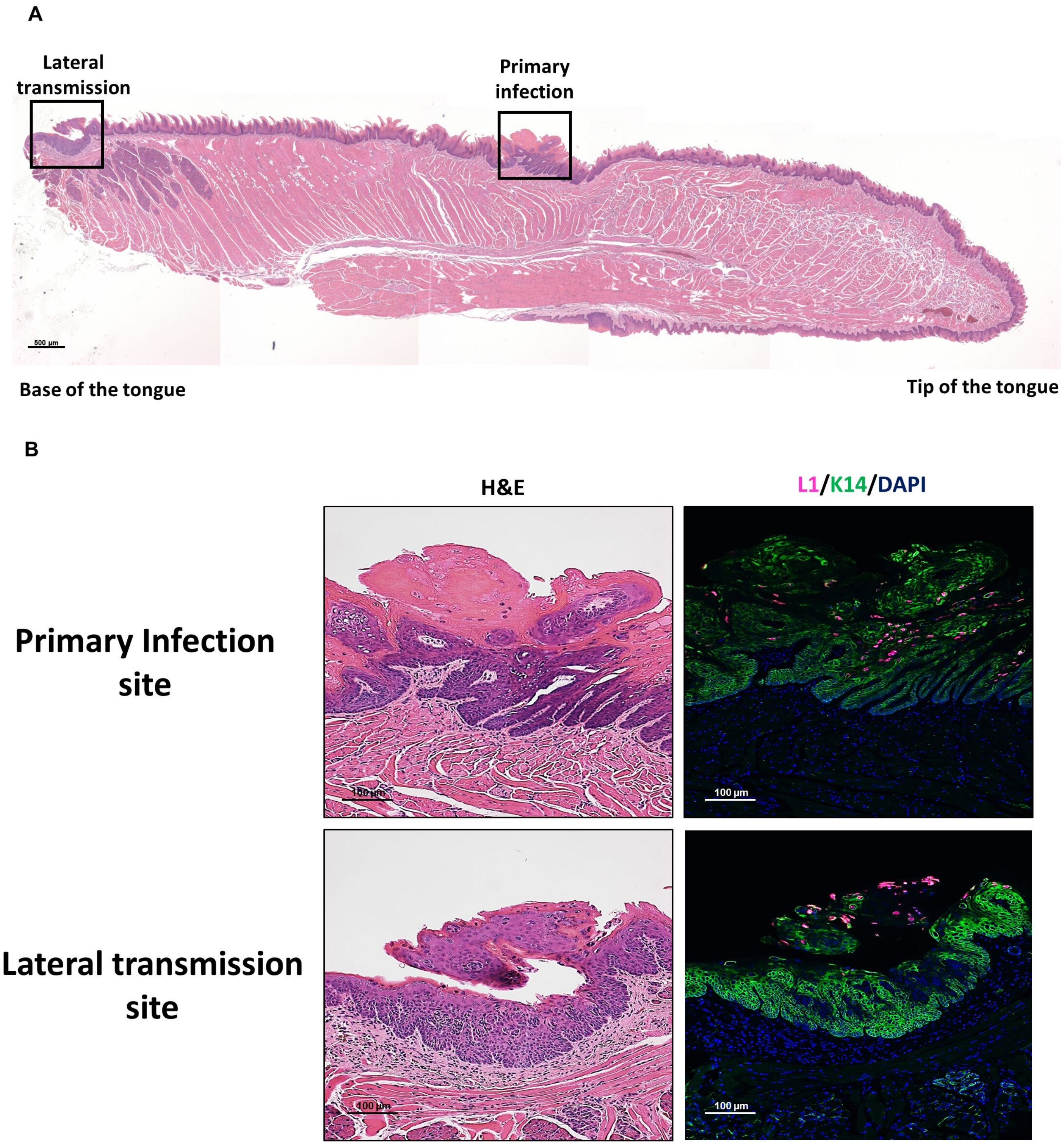
A
Lateral transmission
Primary infection
500 µm
Base of the tongue
Tip of the tongue
B
H&E
L1/K14/DAPI
Primary Infection site
Lateral transmission site
100 µm
100 µm
100 µm
100 µm
