# Supplementary material for: An Infection-Based Murine Model for Papillomavirus-Associated Head and Neck Cancer
Source: mBio. 2020 May 12;11(3):e00908-20. doi: 10.1128/mBio.00908-20 (PMC7218285; doi:10.1128/mBio.00908-20)
Supplement: TABLE S1 [file mBio.00908-20-st001.pdf]

Supplementary table 1. Disease severities of all experimental *FVB* mice in the study, with or without UV and 4NQO treatment, including those excluded from Table 2.

	n	Gender	Normal	Dysplasia			Invasive carcinoma		
				mild	moderate	severe	Grade 1	Grade 2	Grade 3
Mock	5	2M,3F	5	0	0	0	0	0	0
Mock+ 10 μg/mL 4NQO	8	8M	3	4	1	0	0	0	0
Mock + UV+ 10 μg/mL 4NQO	9	5M,4F	6	2	1	0	0	0	0
MmuPV1	6	4M,2F	6 (3)	0	0	0	0	0	0
MmuPV1+ UVB	5	4M,1F	5 (2)	0	0	0	0	0	0
MmuPV1+ 10 μg/mL 4NQO	5	5F	1 (1)	1 (1)	2	1	0	0	0
MmuPV1+ UV+ 10 μg/mL 4NQO	9	5M,4F	2	4 (1)	0	2	0	1	0
MmuPV1+ UV+ 20 μg/mL 4NQO	12	10M,2F	0	3 (1)	3 (1)	2 (2)	3 (1)	0	1

M: male; F: female. Number in parenthesis indicated that the number of mice detected negative for MmuPV1 E4 transcript by *in situ* hybridization, which were the samples that excluded from Table 2.
