# Supplementary material for: An Infection-Based Murine Model for Papillomavirus-Associated Head and Neck Cancer
Source: mBio. 2020 May 12;11(3):e00908-20. doi: 10.1128/mBio.00908-20 (PMC7218285; doi:10.1128/mBio.00908-20)
Supplement: TABLE S2 [file mBio.00908-20-st002.pdf]

Supplementary Table 2. Summary of all primary antibodies used in this study.

Name	Vendor	Catalog Number	Dilution
anti-BrdU	Calbiochem	203806	1:50
anti-MCM7	NeoMarkers	MS862	1:200
anti-pS6	Cell Signaling	4858	1:100
anti-K14	Covance	PRB-155P	1:1000
anti-K17	gift from Dr. Pierre Coulombe (University of Michigan)		1:1000
anti-MmuPV1 L1	gift from Dr. Chris Buck (National Institution of Health)		1:5000
anti-pERK	Cell Signaling	9101	1:100
